# Identification of gemilukast as a bifunctional molecule with lipid-lowering and anti-inflammatory activities

**DOI:** 10.3389/fimmu.2026.1738104

**Published:** 2026-03-04

**Authors:** Yuanyuan Liu, Yixiao Wang, Rufei Wang, Qingshan Yang, Guojun Pan, Renshuai Zhang

**Affiliations:** 1 Department of Internal Neurology, The First Affiliated Hospital of Jinzhou Medical University, Jinzhou, China; 2 Shandong First Medical University & Shandong Academy of Medical Sciences, Jinan, China; 3 Department of Radiation Oncology, The First Affiliated Hospital of Jinzhou Medical University, Jinzhou, China

**Keywords:** cholesterol absorption, dual-action agent, gemilukast, hyperlipidemia, inflammation

## Abstract

**Background:**

Cardiovascular disease is driven by the interplay between dyslipidemia and chronic inflammation. However, most current therapies mainly focus on lipid lowering, leaving substantial residual inflammatory risk and underscoring the need for agents that can address both dyslipidemia and inflammation to reduce cardiometabolic risk. Here, we evaluated whether gemilukast, a cysteinyl leukotriene receptor antagonist, could serve as a bifunctional agent with lipid-lowering and anti-inflammatory activities.

**Methods:**

Intestinal cholesterol absorption was assessed using mixed-micelle solubilization and Caco-2 uptake assays. Anti-inflammatory activity was evaluated in LPS-stimulated RAW 264.7 macrophages by cytokine production and macrophage polarization, with PI3K/AKT signaling examined by Western blotting. Lipid-lowering and anti-inflammatory effects were further validated in an acute hyperlipidemia rat model and the high-fat diet (HFD) induced hyperlipidemia mouse model.

**Results:**

In Caco-2 cells, gemilukast inhibited cholesterol uptake in a concentration-dependent manner, achieving 51.6% inhibition at 10 μM versus vehicle and showing stronger inhibition than ezetimibe (50 μM). In a mixed-micelle assay, gemilukast reduced micellar cholesterol solubility by 41.5%, supporting impaired intestinal cholesterol incorporation. In LPS-stimulated RAW 264.7 macrophages, gemilukast decreased TNF-α, IL-1β, and IL-6, promoted M1-to-M2 repolarization, and was accompanied by reduced PI3K/AKT phosphorylation. *In vivo*, gemilukast (10 mg/Kg) lowered plasma TC by 14.8% at 2 h after an oral lipid challenge in rats. In HFD-fed mice, gemilukast (10 mg/Kg) reduced TC by 25.9% and decreased circulating pro-inflammatory cytokines (IL-1β by 22.0%, IL-6 by 31.5%, and TNF-α by 36.0%).

**Conclusion:**

Gemilukast exhibits dual lipid-lowering and anti-inflammatory activities. These effects are linked to reduced intestinal cholesterol uptake and macrophage reprogramming with PI3K/AKT signaling modulation. These findings provide proof-of-concept that a clinically developed leukotriene receptor antagonist can be repurposed as an immunometabolic bifunctional scaffold to address dyslipidemia with residual inflammatory risk.

## 1 Introduction

Cardiovascular disease remains the leading global cause of mortality, with recent analyses from the Global Burden of Disease study showing persistently high disease burden across regions despite therapeutic advances (1). Dyslipidemia is recognized as a major risk factor for cardiovascular disease (2–4). However, increasing evidence indicates that cardiovascular disease represents an immunometabolic disorder in which dyslipidemia and chronic inflammation act as co-driving forces contributing to disease progression (5–7). Lowering cholesterol levels alone is insufficient to completely eliminate the risk of cardiovascular disease (8). For example, in pooled analyses of populations receiving cholesterol-lowering therapy, high-sensitivity C-reactive protein (an inflammatory marker) was a stronger predictor of future cardiovascular events and mortality than low-density lipoprotein cholesterol (LDL-C) (9). Similar findings have been observed in patients with well-controlled cholesterol levels, where the persistence of inflammation continues to markedly increase the risk of cardiovascular disease even when LDL-C is aggressively managed (10). Consequently, therapeutic strategies aimed at reducing adverse cardiovascular events have shifted from cholesterol-lowering monotherapy toward approaches that concurrently target dyslipidemia and inflammation (11).

Despite advances in understanding the multifactorial nature of cardiovascular disease, therapeutic interventions remain dominated by cholesterol-lowering medications (12, 13). Currently, cholesterol-lowering agents can be broadly classified into three categories: statins, which inhibit endogenous cholesterol biosynthesis (e.g., lovastatin); PCSK9 inhibitors, which promote cholesterol clearance (e.g., evolocumab); and intestinal cholesterol absorption inhibitors, which block dietary cholesterol uptake (e.g., ezetimibe) (14). Statins are the first-line agents for cholesterol reduction; however, up to 20% of patients are unable to tolerate high-dose statin therapy due to potential adverse effects, and some fail to achieve optimal cholesterol targets despite treatment (15, 16). Moreover, although statins may confer anti-inflammatory benefits beyond lipid lowering, nearly half of patients receiving intensive statin therapy still exhibit residual inflammatory risk and an increased incidence of major cardiovascular events (17, 18). Accordingly, agents with dual lipid-lowering and anti-inflammatory actions could offer a more comprehensive strategy for managing cardiovascular risk. Importantly, although several molecules have been reported to possess both lipid-lowering and anti-inflammatory activities, whether they can concurrently modulate intestinal cholesterol absorption and macrophage-driven inflammation remains insufficiently defined (19).

Gemilukast is an orally active cysteinyl leukotriene receptor antagonist initially developed for asthma (20, 21). Gemilukast contains lipophilic aromatic rings and two hydrophilic carboxyl groups, rendering it a prototypical amphipathic compound. This class of compounds holds promise for reducing plasma cholesterol by interfering with the intestinal uptake of dietary cholesterol (22). For example, in our previous study, we found that similar amphipathic molecules inhibited intestinal absorption of dietary cholesterol by reducing its solubility within bile salt-containing mixed micelles, thereby exerting a cholesterol-lowering effect (23). Furthermore, because cysteinyl leukotriene signaling orchestrates inflammatory responses in vascular and immune cells, leukotriene receptor antagonism represents a plausible anti-inflammatory strategy (24, 25). For example, another cysteinyl leukotriene receptor antagonist, montelukast, has been reported to exert anti-inflammatory effects by promoting macrophage polarization (26). These features suggest that gemilukast might serve as a bifunctional agent, combining lipid-lowering and anti-inflammatory properties. Therefore, gemilukast represents a pragmatic repurposing candidate to explore bifunctional lipid-lowering and anti-inflammatory activities with potential relevance to cardiometabolic risk management. Nevertheless, evidence regarding gemilukast’s lipid-lowering and anti-inflammatory effects and the associated mechanisms has not been systematically evaluated.

In the current study, we therefore hypothesized that gemilukast exerts both lipid-lowering and anti-inflammatory activities. We examined its effects on intestinal cholesterol uptake in Caco-2 cells and evaluated its anti-inflammatory activity and macrophage polarization in RAW 264.7 macrophages, followed by mechanistic studies to delineate the basis of both activities. Furthermore, we validated the lipid-lowering activity of gemilukast in both acute and chronic hyperlipidemia models and confirmed its ability to effectively suppress inflammation associated with hyperlipidemia. Collectively, this work introduces gemilukast as a proof-of-concept dual-action scaffold and offers a potential repurposing avenue for cardiometabolic risk management.

## 2 Materials and methods

### 2.1 Chemicals and reagents

Gemilukast, 22-(N-(7-nitrobenz-2-oxa-1,3-diazol-4-yl) amino)-23,24-bisnor-5-cholen-3β-ol (NBD-cholesterol), taurocholate and Filipin were purchased from J&K Scientific. All antibodies were purchased from Proteintech (Wuhan, Hubei, China). Commercial kits for total cholesterol (TC), triglycerides (TG), LDL-cholesterol (LDL-C), HDL-cholesterol (HDL-C), alanine aminotransferase (ALT) and aspartate aminotransferase (AST) were purchased from Nanjing Jiancheng Bioengineering Institute. Enzyme-linked immunosorbent assay (ELISA) kits for TNF-α, IL-1β, and IL-6 were purchased from Boster Biological Technology Co., Ltd. The primers of IL-1β, IL-6, TNF-α, Arg-1, IL-10 and housekeeping gene of GAPDH were designed by Huayue Gene (Wuhan, China). The high-fat diet (HFD) feed, consisting of 77.6% maintenance feed, 10% lard, 10% yolk powder, 2% cholesterol, and 0.2% bile salt, was purchased from HuaFuKang Biotechnology (Beijing, China).

### 2.2 Cell uptake assay of cholesterol

The cellular cholesterol-uptake assay was performed following the procedure of Jia et al. (23). NBD-cholesterol/taurocholate mixed micelles were prepared as described by Johnson et al. (27). Prior to uptake measurements, Caco-2 cells were cultured for 24 h in medium supplemented with lipoprotein-deficient serum. Cells were then rinsed three times with phosphate-buffered saline (PBS), incubated with gemilukast for 4 h, and subsequently exposed to NBD-cholesterol/taurocholate micelles for 1 h. After three additional PBS washes to remove non-internalized fluorescent cholesterol, fluorescence was recorded at 485 nm (excitation) and 535 nm (emission). Ezetimibe was included as a positive control. Vehicle control contained the same solvent concentration as the drug-treated groups. Fluorescence signals were background-subtracted and normalized to the vehicle control (set as 100%) for presentation.

### 2.3 Cholesterol-solubility assay

The micellar cholesterol-solubility assay was adapted from Su et al. (28). Briefly, a bile salt micellar solution was prepared by combining 0.4 mM cholesterol, 10 mM sodium taurocholate, 132 mM NaCl, 1 mM oleic acid, and 15 mM sodium phosphate buffer (pH 6.8), followed by sonication until clarity. The suspension was equilibrated at 37°C for 24 h. Aliquots (1.0 mL each) of the micellar solution were then supplemented with gemilukast at the indicated concentrations (vehicle matched across groups), shaken in a water bath at 37°C for 2 h, and centrifuged at 15,000 rpm for 20 min. The supernatant was carefully collected without disturbing the pellet, and micellar cholesterol was quantified using an enzymatic assay according to the manufacturer’s instructions.

### 2.4 Filipin staining

A 5 mg/mL Filipin stock solution was freshly prepared as described previously (23). The cholesterol-depleting medium was formulated according to Ge et al. (29). Caco-2 cells grown in confocal chambers were incubated in the depleting medium for 1 h, then treated with the indicated concentrations of gemilukast for an additional 1 h. Cells were rinsed three times with PBS and fixed in 4% formaldehyde. After two further PBS washes, cholesterol was visualized by staining with Filipin (50 μg/mL, 30 min, room temperature, protected from light), followed by three PBS washes. Images were acquired on a Nikon confocal microscope, with Filipin fluorescence displayed using red pseudo-color. For imaging, acquisition settings were kept constant across groups within each experiment. Fluorescence intensity was quantified using ImageJ by measuring mean fluorescence intensity per field (background-subtracted) from at least three randomly selected fields per condition.

### 2.5 Cytokines testing and PCR

Cytokines in culture supernatants were quantified using ELISA kits. For quantitative PCR, RAW 264.7 cells (5 × 10^4^ cells/mL) were stimulated with LPS (1.0 μg/mL) for 24 h. Total RNA was isolated with a commercial extraction kit and reverse-transcribed to cDNA. qPCR was performed with GAPDH as the endogenous control under the following cycling conditions: 95°C for 15 s, 60°C for 30 s, and 72°C for 30 s, for 40 cycles. Primer sequences were designed using Primer 5.0 software (**Supplementary Table 1**). Each qPCR reaction was run in technical triplicates, and relative expression was calculated by the 2^-ΔΔCt^ method normalized to GAPDH.

### 2.6 Macrophage phenotype transition

Immunofluorescence staining was adapted from Yang et al. (30). Briefly, RAW 264.7 cells were seeded on coverslips in 24-well plates and stimulated with LPS (1.0 μg/mL) for 48 h. Cells were then treated with gemilukast (10 µM) for an additional 24 h and fixed in 4% paraformaldehyde. Primary antibodies against CD68 (1:100), iNOS (1:50), or CD206 (1:50) were applied at 4°C overnight, followed by incubation with the corresponding fluorescent secondary antibodies for 30 min. Nuclei were counterstained with DAPI and coverslips were mounted with antifade medium. Mean fluorescence intensity of iNOS and CD206 was quantified in ImageJ under identical acquisition settings for all groups.

### 2.7 Western blot assay

Proteins were extracted and quantified by BCA assay. For each gel lane, 5-10 μg of total protein was loaded. After electrophoresis, proteins were transferred to membranes at a constant current of 250 mA, with transfer time adjusted to the molecular weight of the target. Membranes were then blocked for 15–25 min prior to antibody incubation. Membranes were incubated with primary antibodies at 4°C overnight, followed by incubation with HRP-conjugated secondary antibodies at room temperature. Protein bands were visualized using enhanced chemiluminescence. Signals were captured using an Amersham ImageQuant™ 800 imager (Cytiva). Band intensities were quantified by densitometry using ImageJ; phosphorylated proteins were normalized to the corresponding total proteins, and total proteins were normalized to GAPDH as indicated.

### 2.8 Animal studies

Animal experiments were performed under approval from the IACUC of Shandong First Medical University (W202403010164) in accordance with the NRC Guide for the Care and Use of Laboratory Animals. Male C57BL/6J mice and Sprague-Dawley (SD) rats (8 weeks) were purchased from Beijing Vital River (Beijing, China). After a 7-day acclimation at 25 ± 2°C with a 12:12 h light-dark cycle, animals were assigned to acute or long-term experimental protocols. At study endpoints, animals were humanely euthanized by cervical dislocation in accordance with institutional Standard Operating Procedures (SOPs). Trained personnel performed the procedure, and death was confirmed by cardiopulmonary arrest and fixed, dilated pupils.

In the acute lipid-absorption study, SD rats were randomly allocated to four groups (n = 5). A lipid emulsion was prepared from yolk powder and olive oil (1 g:10 mL, w/v) and sonicated for 10 min. Animals were fasted for 12 h before dosing. Experimental groups received the lipid emulsion by oral gavage (10 mL/kg); gemilukast was co-administered in the emulsion at 5 mg/kg or 10 mg/kg. Controls received an equal volume of water. Blood was collected at 1-h intervals for determination of plasma TC and TG.

In the long-term experiment, C57BL/6J mice were randomized into five groups (n = 5): control, high-fat diet (HFD), positive control (lovastatin), and two gemilukast groups (5 mg/kg/day and 10 mg/kg/day). Controls received standard chow, whereas the remaining groups were maintained on HFD. Lovastatin and gemilukast were administered once daily by oral gavage in the morning. Body weight was recorded weekly, and food intake was monitored every 2 days. At study termination, mice were euthanized and blood and tissues were collected for analysis; liver samples were processed for protein extraction and histopathology. Aspartate aminotransferase (AST) and alanine aminotransferase (ALT) were measured using commercial assay kits following the manufacturer’s instructions. Fixed liver specimens were paraffin-embedded and sectioned at 3 µm. Sections were stained with hematoxylin and eosin (H&E) for histopathological evaluation of hepatic injury. Histopathological evaluation was performed in a blinded manner by an experienced investigator who was unaware of group allocation.

### 2.9 Statistical analysis

Data are presented as mean ± standard error of the mean. Group comparisons were performed by one-way analysis of variance (ANOVA). A two-sided P value < 0.05 was considered statistically significant. Analyses were conducted using GraphPad Prism 8.0 (GraphPad Software, San Diego, CA, USA).

## 3 Results

### 3.1 Gemilukast inhibits cholesterol uptake

**Figure 1:**
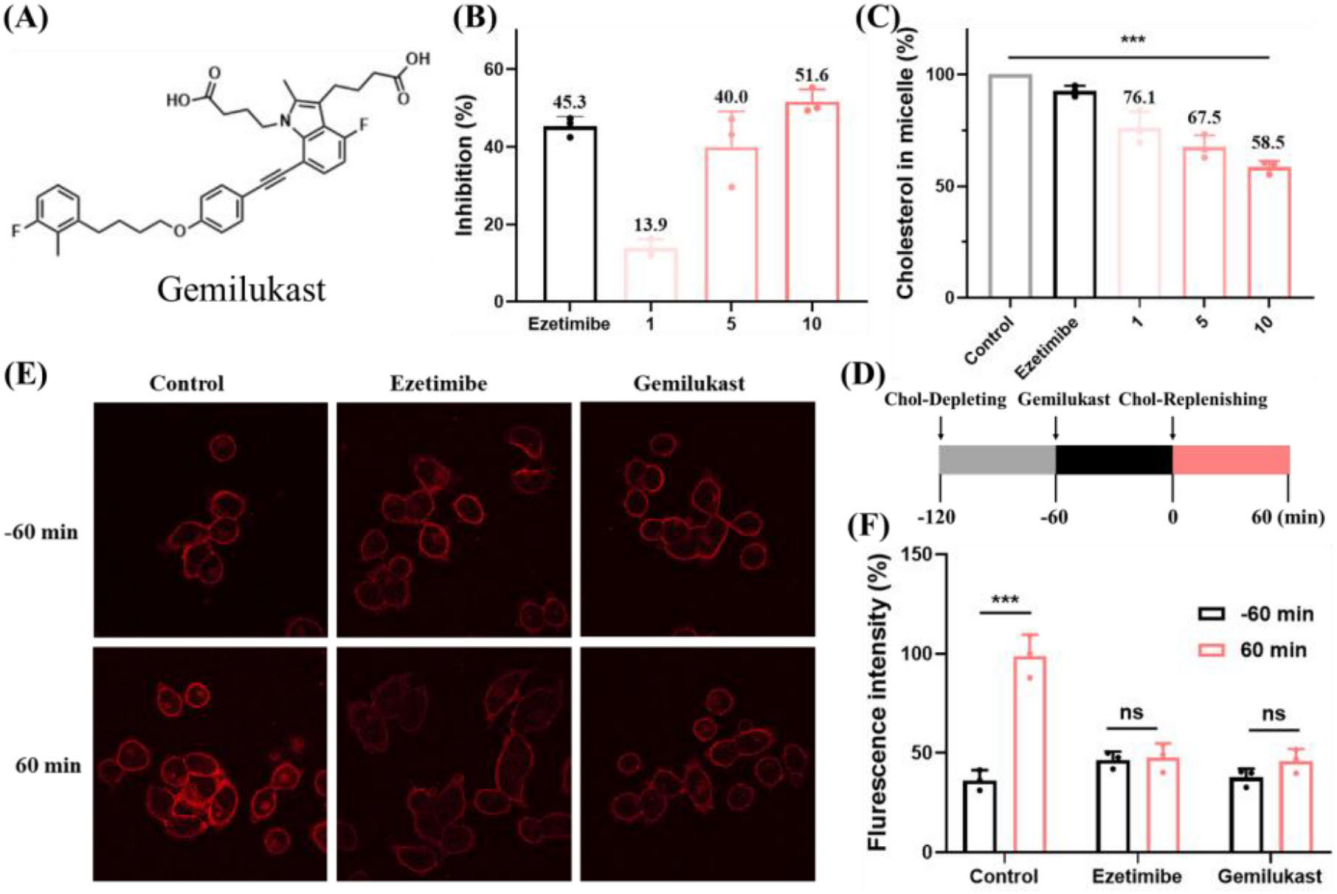
Gemilukast inhibits cholesterol uptake. **(A)** Chemical structure of gemilukast. **(B)** Inhibition of cholesterol uptake in Caco-2 cells by gemilukast (1, 5, 10 μM), with ezetimibe (50 μM) serving as a reference inhibitor. **(C)** Impact of gemilukast (1, 5, 10 μM) on the micellar solubilization of cholesterol (expressed as % of control). **(D)** Experimental workflow for Caco-2 treatment. Chol: cholesterol. **(E)** Representative fluorescence images of Caco-2 cells treated as in panel **(D)**, Scale bar = 20 μm. **(F)** Quantification of intracellular cholesterol fluorescence. Dots indicate independent replicates, n = 3. ^***^P < 0.001; ns, not significant.

The chemical structure of gemilukast is shown in **Figure 1A**. Before evaluating the cholesterol-lowering and anti-inflammatory activities of gemilukast, its cytotoxicity was first assessed by cell viability assays in Caco-2 cells (cholesterol uptake model) and RAW 264.7 macrophages (inflammation model). As shown in **Supplementary Figure 1**, gemilukast exhibited no significant cytotoxicity at a concentration of 10 µM, with more than 90% of cells remaining viable. Therefore, a concentration of 10 µM was selected for subsequent experiments. Cholesterol uptake was evaluated in Caco-2 cells using the NBD-cholesterol assay, a commonly used method for assessing cholesterol absorption inhibitors (31). Gemilukast dose-dependently inhibited cholesterol uptake and achieved 51.6% inhibition at 10 μM versus vehicle group, exceeding the effect of ezetimibe (50 μM), an FDA-approved cholesterol absorption inhibitor (**Figure 1B**). Because micellar solubilization is required for cellular cholesterol uptake, we next examined whether gemilukast affects cholesterol solubilization in mixed micelles using a cholesterol-incorporation assay. The addition of gemilukast led to a distinct dose-dependent inhibition of cholesterol incorporation, as demonstrated in **Figure 1C**. Compared with micelles without gemilukast (control group), the cholesterol content in the micellar phase decreased by 41.5%, suggesting that gemilukast interfered with cholesterol incorporation into mixed micelles.

Filipin staining is widely used to visualize intracellular cholesterol (32). Here, the Filipin staining assay was also employed to further assess cellular cholesterol uptake (**Figures 1D–F**). The experimental sequence is illustrated in **Figure 1D**. Briefly, Caco-2 cells were first incubated for 1 h in a cholesterol-depleting medium to reduce intracellular sterol pools, followed by a 60 min exposure to gemilukast. Subsequently, a cholesterol-replenishing medium was applied. Confocal images were collected immediately before replenishment (-60 min) and after 60 min of replenishment. As shown in **Figure 1E**, 60 min in the depleting medium resulted in minimal Filipin fluorescence in Caco-2 cells. In contrast, 60 min of incubation with the replenishing medium markedly increased the cellular Filipin signal. Notably, gemilukast attenuated this fluorescence recovery, indicating effective inhibition of cholesterol uptake. Quantitative fluorescence analysis (**Figure 1F**) was concordant with these observations. Collectively, these findings support a mechanism whereby gemilukast limits cholesterol incorporation into mixed micelles, thereby restricting subsequent uptake by Caco-2 cells.

### 3.2 Gemilukast effectively alleviates inflammation

**Figure 2:**
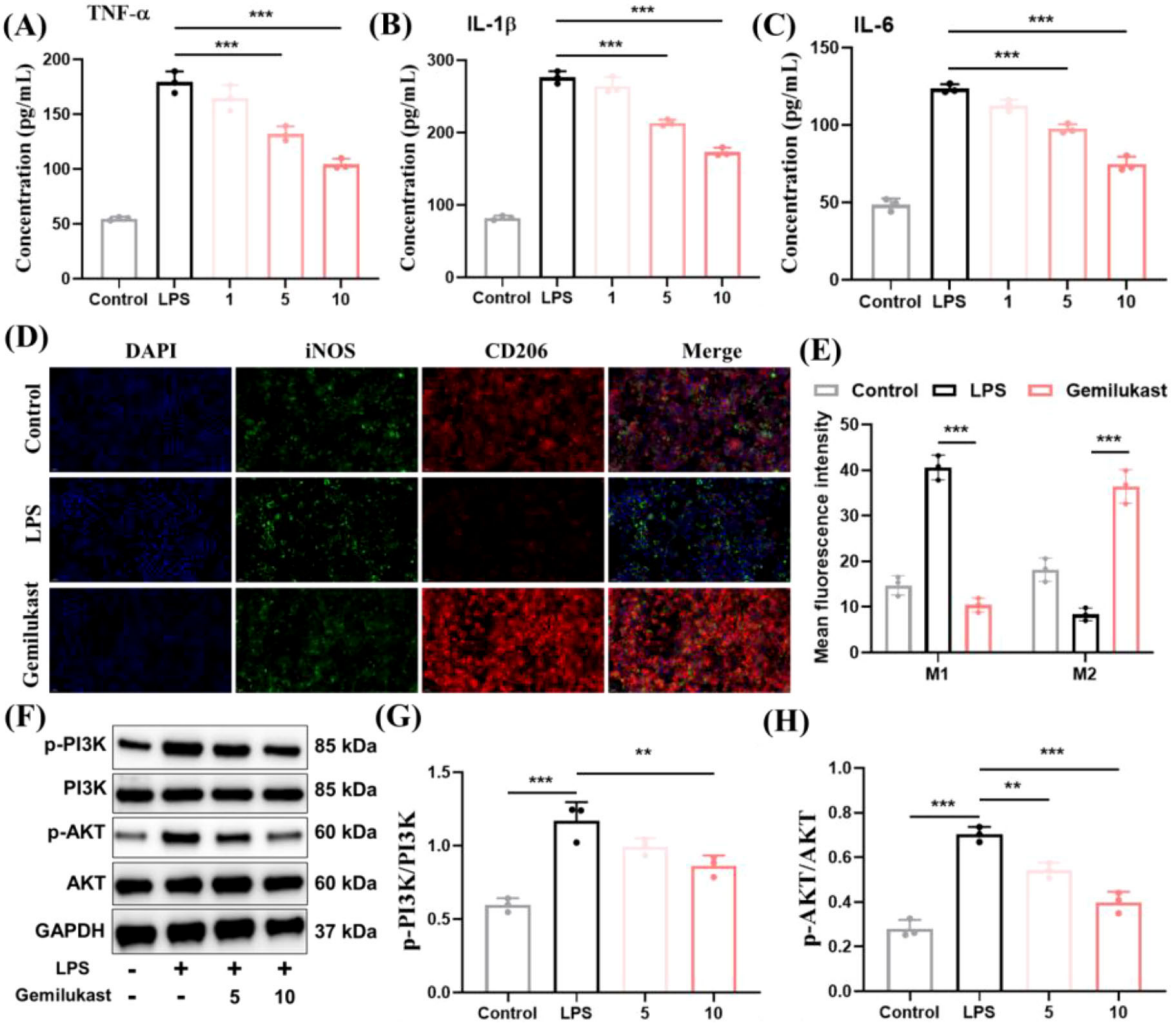
Gemilukast attenuates LPS-induced inflammatory responses in RAW 264.7 macrophages. **(A–C)** TNF-α, IL-1β, and IL-6 levels in RAW 264.7 cells stimulated with LPS and treated with gemilukast at the indicated concentrations (1, 5, 10 μM). **(D)** Immunofluorescence staining of iNOS (green), CD206 (red), and nuclei (blue) on RAW 264.7 macrophages without or with treatment of gemilukast. Scale bar = 50 μm. **(E)** Mean fluorescence intensity of iNOS (M1) and CD206 (M2). **(F)** Western blot of PI3K/AKT signaling pathways in RAW 264.7 cells under the indicated treatments (24 h). **(G, H)** The statistics of protein levels of results from **(F)**. ^**^P < 0.01, ^***^P < 0.001.

Gemilukast is a cysteinyl leukotriene receptor antagonist with antiasthmatic potential (21). To date, research on gemilukast has focused largely on target-level pharmacology, whereas studies addressing its anti-inflammatory activity and mechanisms at the cellular and *in vivo* levels remain limited. As shown in **Figures 2A–C**, cytokine analysis revealed that gemilukast significantly reduced the levels of pro-inflammatory cytokines TNF-α, IL-1β, and IL-6 at concentrations of 5 µM and 10 µM, indicating its anti-inflammatory activity. It has been demonstrated that macrophages play a crucial role in the initiation and progression of inflammation (33). M1 macrophages secrete pro-inflammatory cytokines that amplify inflammatory responses, whereas M2 macrophages release anti-inflammatory mediators that promote inflammation resolution (34). Therefore, promoting the polarization of macrophages from the M1 to the M2 phenotype represents an effective anti-inflammatory strategy. To further elucidate the anti-inflammatory mechanism of gemilukast, we investigated gemilukast’s effects on macrophage polarization. LPS-treated RAW 264.7 cells, exhibiting a typical M1 pro-inflammatory phenotype, were included as the positive control. Immunofluorescence staining was performed to analyze the expression of the M1 marker iNOS and the M2 marker CD206. Following gemilukast exposure, iNOS levels were reduced and CD206 staining was enhanced (**Figure 2D**), suggesting effective repolarization of macrophages from M1 to M2 type. In line with imaging data, fluorescence quantification indicated a decrease in M1 macrophages and an increase in M2 macrophages following gemilukast treatment (**Figure 2E**). To further confirm these findings, real-time polymerase chain reaction (RT-PCR) was conducted to evaluate the mRNA expression levels of macrophage markers, including IL-1β, IL-6, TNF-α, Arg-1, and IL-10. As shown in **Supplementary Figure 2**, compared with untreated cells, gemilukast significantly downregulated the expression of M1 markers (IL-1β, IL-6 and TNF-α) while upregulating M2 markers (Arg-1 and IL-10). These results indicate that gemilukast promotes the polarization of macrophages from the M1 to the M2 phenotype.

The PI3K/AKT signaling pathway is involved in the activation of M1 macrophages and represents a classic inflammatory signaling cascade that plays a crucial role in inflammatory diseases (35, 36). To investigate the regulatory effect of gemilukast on the PI3K/AKT signaling pathway, we examined the phosphorylation levels of PI3K and AKT (**Figures 2F–H**). Compared with the control group, LPS treatment significantly increased the ratios of *p*-PI3K/PI3K and *p*-AKT/AKT, whereas gemilukast markedly reduced these phosphorylation ratios relative to the LPS group, indicating that gemilukast inhibited the activation of the PI3K/AKT signaling pathway. Collectively, the current results indicate that gemilukast decreases M1 macrophage formation through modulation of PI3K/AKT signaling, leading to reduced inflammation.

### 3.3 Gemilukast exhibits significant lipid-lowering activity *in vivo*

**Figure 3:**
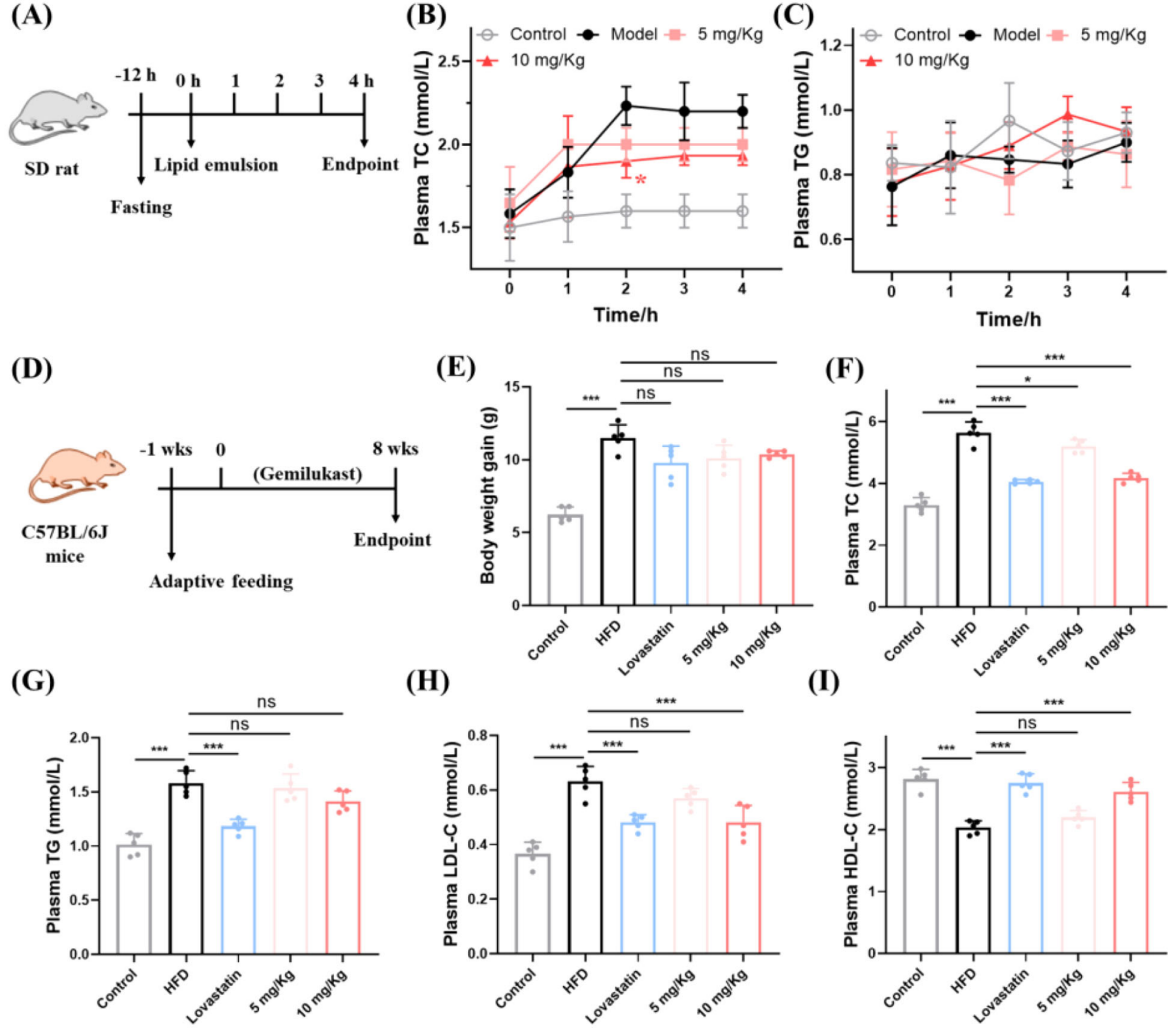
Gemilukast exhibits significant lipid-lowering activity *in vivo*. **(A)** Schedule of acute hyperlipidemia SD rat treatment. **(B)** TC level in acute hyperlipidemia SD rats. **(C)** TG level in acute hyperlipidemia SD rats. **(D)** Schedule of long-term hyperlipidemia treatment. **(E)** Body weight gain in mice during the HFD feeding period with the indicated treatments. **(F-I)** Plasma levels of TC, TG, LDL-C and HDL-C. Dots indicate individual animals (n = 5). ^*^P < 0.05, ^***^P < 0.001; ns, not significant.

To assess whether gemilukast impairs intestinal absorption of cholesterol and triglycerides *in vivo*, we measured plasma total cholesterol (TC) and triglycerides (TG) at serial time points in a rat model of acute hyperlipidemia (**Figures 3A–C**). Following oral lipid emulsion, TC in the model group rose markedly relative to the control group, whereas gemilukast administration significantly lowered TC at 2 h (**Figure 3B**). At 10 mg/kg, gemilukast reduced plasma TC by 14.8% at the 2 h time point compared with the model group, indicating that gemilukast may interfere with the intestinal uptake of cholesterol. For TG, there were no significant differences between the high-dose gemilukast and control groups throughout the 4-hour assessment (**Figure 3C**), which is plausibly explained by the requirement for luminal hydrolysis and subsequent re-esterification of TG prior to systemic appearance, a process that likely delays detectable changes.

The hypolipidemic activity of gemilukast was further assessed in a C57BL/6J hyperlipidemia model. The schedule of long-term protocol is presented in **Figure 3D**. The FDA-approved lipid-lowering drug lovastatin was used as the positive control. The body weight of each mouse was monitored and documented once per week throughout the study period. The concentrations of TG, TC, LDL-cholesterol (LDL-C) and HDL-cholesterol (HDL-C) were measured at the conclusion of the experiment. The overall body weight trend is shown in **Supplementary Figures 3A**, and the weight gain pattern is presented in **Figure 3E**. The HFD group exhibited a significantly greater body weight gain than the control group. However, with similar food consumption among groups (**Supplementary Figure 3B**), the body weight of gemilukast-treated mice did not differ significantly from HFD group (**Figure 3E**), suggesting that gemilukast did not affect body weight. In addition, as shown in **Figures 3F–I**, the effects of gemilukast on HFD-induced plasma lipid levels were investigated. HFD-induced robust increases in plasma TG and TC, while gemilukast significantly decreased TC compared with HFD groups (by 25.9%) but had no impact on TG (**Figures 3F, G**). Furthermore, although low-dose gemilukast had no effect on LDL-C and HDL-C, high-dose gemilukast significantly decreased LDL-C and markedly increased HDL-C (**Figures 3H, I**). Collectively, *in vivo* studies show that gemilukast not only significantly lowers TC in an acute hyperlipidemia model, but also improves the lipoprotein profile through reducing LDL-C and increasing HDL-C in long-term hyperlipidemia model, without affecting body weight.

### 3.4 Gemilukast exhibits significant anti-inflammatory activity *in vivo*

**Figure 4:**
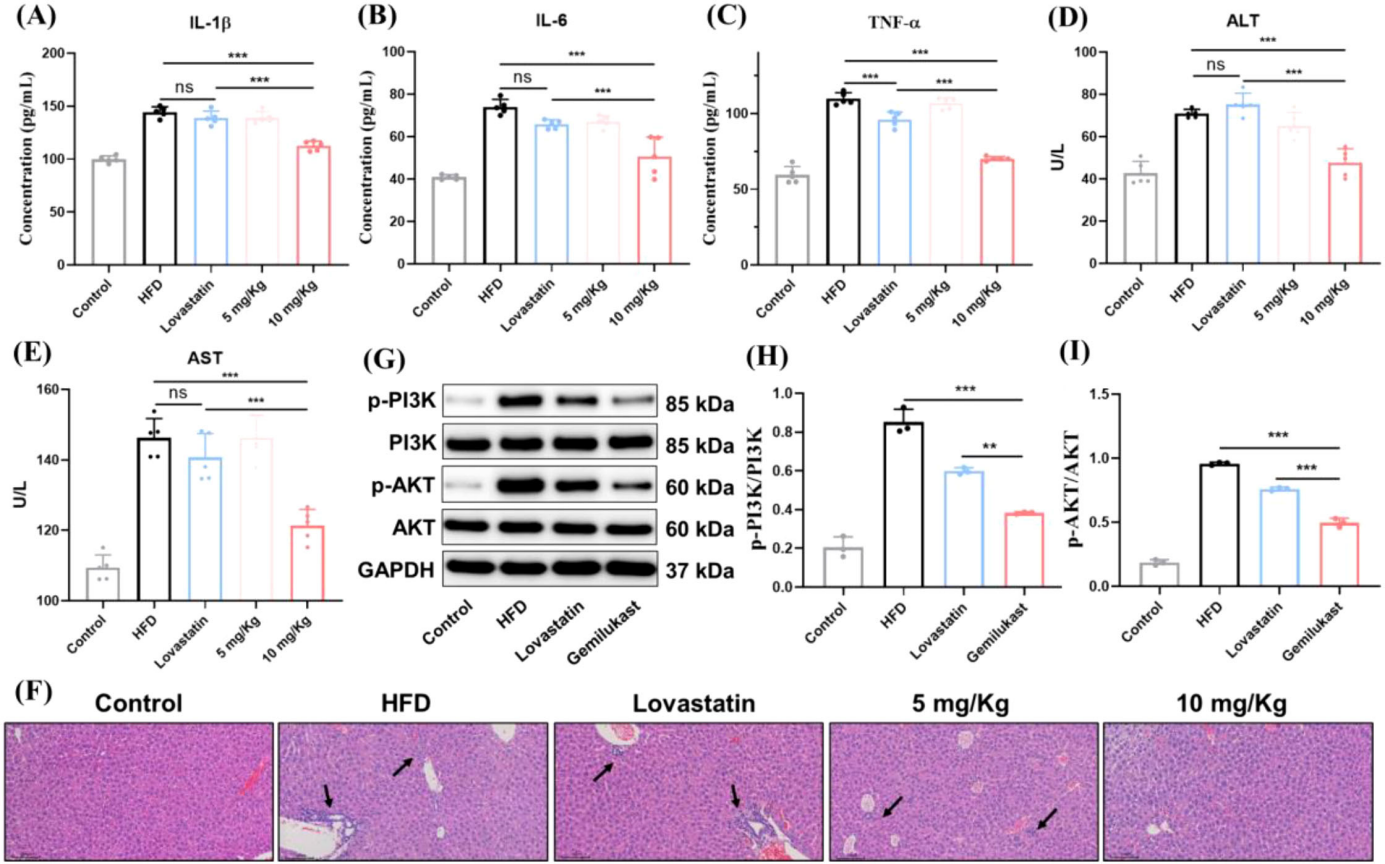
Gemilukast exhibits significant anti-inflammatory activity in HFD-fed mice. **(A-C)** Serum levels of IL-1β, IL-6, and TNF-α measured by ELISA in control, HFD, lovastatin, and gemilukast-treated mice (5 or 10 mg/kg). **(D, E)** Serum ALT and AST levels as indicators of hepatic safety. **(F)** Representative H&E-stained liver sections after the indicated treatments (arrows indicate pathological changes). Scale bar = 100 μm. **(G)** Representative immunoblots of PI3K/AKT pathway proteins in liver tissues (gemilukast, 10 mg/kg). **(H, I)** The statistics of protein levels of results from G. Dots indicate individual animals [**(A–E)** n = 5; **(H, I)** n = 3]. ^*^P < 0.05, ^**^P < 0.01, ^***^P < 0.001; ns, not significant.

Inflammation frequently accompanies hyperlipidemia induced by HFD (37). To evaluate the anti-inflammatory effects of gemilukast *in vivo*, the levels of inflammatory cytokines were measured using ELISA. The results show that HFD feeding resulted in higher serum concentrations of IL-1β, IL-6 and TNF-α. However, high-dose gemilukast treatment significantly reduced the levels of these pro-inflammatory factors (**Figures 4A–C**). Specifically, at 10 mg/kg, gemilukast reduced serum IL-1β by 22.0%, IL-6 by 31.5%, and TNF-α by 36.0% compared with the HFD group. In addition, lovastatin lowered plasma TNF-α compared with HFD, but had no significant effect on IL-1β or IL-6, suggesting that its anti-inflammatory efficacy is inferior to that of gemilukast. Extensive evidence shows that hyperlipidemia can cause liver damage by provoking chronic inflammatory responses (38). Considering alanine transaminase (ALT) and aspartate aminotransferase (AST) are the sensitive markers of liver injury, in this study, the serum levels of liver enzymes ALT and AST were measured to evaluate the hepatotoxicity after treatment (**Figures 4D, E**). The HFD group showed dramatic increases in serum ALT and AST activities compared with the control group, while treatment with high-dose gemilukast significantly decreased the activities of serum ALT and AST. In addition, the hepatic enzyme activities in the lovastatin-treated group showed no significant difference compared with the HFD group, which is consistent with previous reports that statins may induce hepatotoxicity (39). Moreover, H&E staining showed that high-dose gemilukast markedly reduced the inflammatory infiltrates associated with hyperlipidemia, while mild inflammation persisted after lovastatin treatment (**Figure 4F**). To assess gemilukast’s anti-inflammatory mechanism *in vivo*, hepatic PI3K and AKT phosphorylation were examined (**Figures 4G–I**), which has been demonstrated to play an important role in inflammatory diseases. The HFD group showed significant elevation in the ratios of *p*-PI3K/PI3K and *p*-AKT/AKT compared with the control group. Interestingly, compared with the HFD group, gemilukast (10 mg/kg) produced a statistically significant inhibition in the ratios of hepatic *p*-PI3K/PI3K and *p*-AKT/AKT. Taken together, these findings indicate that gemilukast exhibits significant anti-inflammatory activity with lower hepatotoxicity than lovastatin *in vivo*, and the anti-inflammatory activity of gemilukast is, at least in part, mediated by the PI3K/AKT signaling pathway.

## 4 Discussion

**Figure 5:**
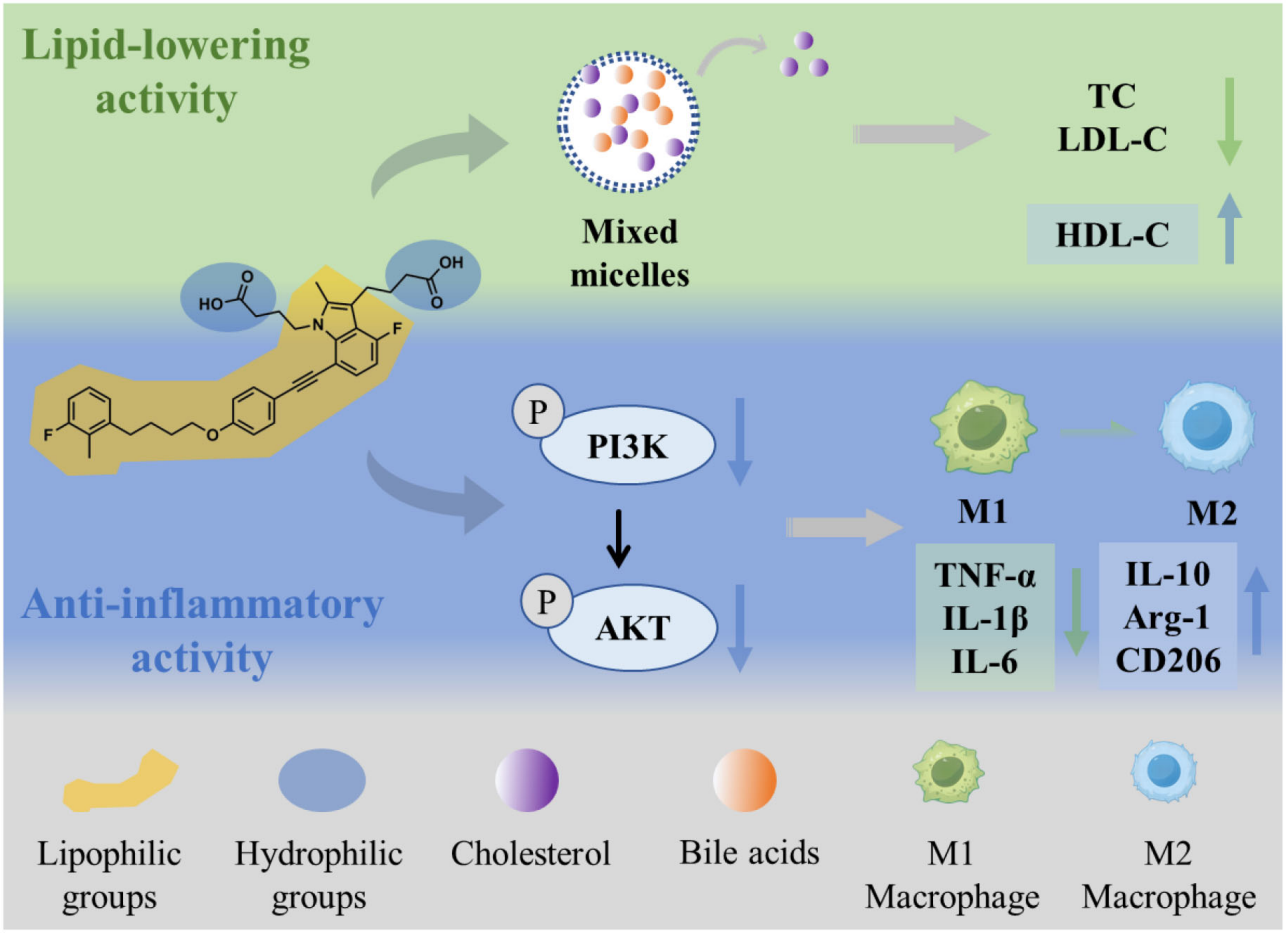
Mechanism of the lipid-lowering and anti-inflammatory activities of gemilukast.

It is well established that dyslipidemia and inflammation are key drivers of cardiovascular disease (19). Extensive research further shows that sustained dyslipidemia provokes chronic inflammation and promotes metabolic diseases such as hyperlipidemia, insulin resistance, and type 2 diabetes (40, 41). However, current management of dyslipidemia-related conditions (e.g., atherosclerosis) relies predominantly on lipid-lowering therapy (e.g., statins), with comparatively limited emphasis on mitigating chronic inflammation. Developing drugs that combine lipid-lowering and anti-inflammatory activities represents an effective strategy for treating dyslipidemia-related diseases. Herein, we identify gemilukast as a candidate molecule with both lipid-lowering and anti-inflammatory activities, and elucidate the molecular mechanisms underlying these dual effects in both *in vitro* and *in vivo* models of hyperlipidemia (**Figure 5**). Our study demonstrates that gemilukast inhibits cholesterol absorption by diminishing solubilization of cholesterol in mixed micelles, and exerts anti-inflammatory effects by suppressing PI3K/AKT signaling to promote macrophage polarization toward the M2 phenotype.

Plasma cholesterol is the principal parameter in the treatment of hyperlipidemia-related cardiovascular diseases (42). Endogenous biosynthesis is the principal source of cholesterol, yet intestinal absorption constitutes a critical additional input to the body’s cholesterol pool (42). The FDA-approved lipid-lowering drug ezetimibe exerts its effect by inhibiting intestinal cholesterol absorption. In addition, although the first-line lipid-lowering agents (e.g. statins) act by inhibiting cholesterol biosynthesis, more than 20% of patients are either unresponsive to statins or intolerant of them (43). Therefore, suppression of intestinal cholesterol absorption is an effective approach for plasma cholesterol reduction, notably in patients who cannot tolerate high-dose statins or who fail to achieve treatment goals on statins (44). Cholesterol absorption is typically regarded as active transport, for which Niemann-Pick C1-like 1 (NPC1L1) serves as the major transporter (29). Given the lipophilicity of cholesterol and the aqueous environment of membrane transport proteins (e.g. NPC1L1), cholesterol is first packaged into mixed micelles with bile salts, enabling subsequent interaction with the transporter (23). Therefore, mixed micelles of free cholesterol and bile acids constitute an essential intermediate for intestinal cholesterol uptake (45). Accordingly, interference with this micellar assembly is expected to diminish cholesterol absorption. Numerous agents have been shown to inhibit absorption by lowering the solubility of cholesterol within micelles (46). Gemilukast contains lipophilic aromatic rings and two hydrophilic carboxyl groups, conferring amphipathic character, which could enable it to disrupt the assembly/stability of cholesterol micelles. Although the lipid-lowering activity of gemilukast has not been previously reported, amphipathic molecules of this type have been demonstrated to suppress cholesterol uptake (23). In the present study, gemilukast exhibited significantly greater inhibition of cholesterol uptake than ezetimibe (**Figure 1B**), an FDA-approved inhibitor of intestinal cholesterol absorption (14). In addition, gemilukast significantly reduced the solubility of cholesterol in mixed micelles (**Figure 1C**), consistent with our hypothesis and providing a mechanistic basis for its inhibition of cholesterol uptake. Moreover, our research demonstrated that gemilukast inhibits intestinal cholesterol absorption in two animal models (**Figure 3**). In the acute hyperlipidemia model, gemilukast significantly reduced plasma TC, providing direct evidence of impaired intestinal cholesterol uptake. In the HFD-induced model, gemilukast likewise lowered TC and favorably remodeled the lipoprotein profile (decreasing LDL-C and increasing HDL-C) without affecting body weight. However, the limitations of the present study regarding the lipid-lowering effects of gemilukast should also be considered. When intestinal cholesterol absorption is inhibited, excess cholesterol may be eliminated via fecal excretion. However, fecal sterol output was not evaluated here. Future work should incorporate small-intestinal segment specific experiments to verify the site of action of gemilukast and to furnish direct *in vivo* mechanistic evidence.

Besides dyslipidemia, inflammation plays an important role in the pathogenesis of hyperlipidemia-related cardiovascular disease (e.g. atherosclerosis) (47). Inflammatory biomarkers have been demonstrated to predict cardiovascular disease, which are independent of traditional risk factors (48). Given that first-line therapies for cardiovascular disease, exemplified by statins, primarily lower lipid levels, the relative contributions of inflammation and dyslipidemia to future cardiovascular risk might change after statin treatment, which in turn can influence the choice of adjunctive therapies (9). Therefore, identifying lipid-lowering drugs with anti-inflammatory properties is crucial for the treatment of cardiovascular disease. Gemilukast is a cysteinyl leukotriene receptor antagonist that, in principle, suppresses leukotriene-mediated inflammatory signaling by blocking ligand-receptor interactions (21). Although leukotriene receptor antagonists, such as montelukast, have documented anti-inflammatory activity, the anti-inflammatory effects of gemilukast have not yet been reported (49). Notably, despite both being leukotriene receptor antagonists, gemilukast and montelukast have profoundly different structures, thus the anti-inflammatory activity of gemilukast remains to be investigated.

In the present study, the lipid-lowering activity of gemilukast has been confirmed (**Figures 1**, **3**). Furthermore, we demonstrated its anti-inflammatory activity and elucidated the underlying mechanism (**Figures 2**, **4**). The current study extends the pharmacological profile of gemilukast beyond receptor-level antagonism by demonstrating anti-inflammatory activity in cell-based and *in vivo* settings. The coordinated decrease in TNF-α, IL-1β, and IL-6, together with a shift from M1 toward M2 macrophages, supports a mechanism centered on macrophage reprogramming. The observed changes in PI3K/AKT phosphorylation are consistent with previous evidence demonstrating that the PI3K/AKT pathway is involved in LPS-induced M1 macrophage activation. However, we did not determine whether gemilukast directly targets the PI3K/AKT pathway; therefore, we interpret the reduced phosphorylation as downstream signaling modulation potentially linked to cysteinyl leukotriene receptor antagonism. Conceptually, these findings suggest that gemilukast may dampen immunometabolic inflammation by interrupting a PI3K/AKT-dependent axis of macrophage polarization. Future work should test causality with pathway-specific gain/loss-of-function tools (e.g., AKT activators or PI3K rescue), confirm pathway dependence in primary human macrophages, and evaluate durability and dose-response *in vivo* to clarify translational potential.

Leukotriene signaling is closely linked to innate immune activation, and cysteinyl leukotriene receptor engagement can shape macrophage inflammatory programs with convergence on downstream pathways such as PI3K/AKT. In this context, leukotriene receptor antagonists have been reported to modulate immune responses beyond airway inflammation. For example, montelukast attenuates neuroinflammation in experimental autoimmune encephalomyelitis by affecting Th17-associated responses, and has also been associated with macrophage polarization in other settings (50). While we did not perform a head-to-head comparison between montelukast and gemilukast in the present study, our data support gemilukast as a bifunctional scaffold that couples macrophage anti-inflammatory reprogramming with inhibition of intestinal cholesterol uptake, providing a cardiometabolic-oriented mechanism distinct from prior montelukast-focused immunological models. Direct comparative studies across matched models and exposures will be valuable in future work to define relative potency and receptor/pathway dependence.

Clinically used lipid-lowering therapies primarily target cholesterol biosynthesis (statins), clearance (PCSK9 inhibitors), or intestinal absorption (ezetimibe). While these agents effectively reduce LDL-C, residual inflammatory risk remains common in a substantial proportion of patients receiving intensive lipid-lowering therapy. Anti-inflammatory strategies may reduce inflammatory burden but do not directly address dyslipidemia, and are not universally applicable due to safety, cost, or patient selection considerations. Although statins exhibit modest anti-inflammatory effects, and some studies suggest that part of their cardiovascular benefit may arise from lipid-independent suppression of vascular inflammation, nearly half of statin-treated patients have residual inflammatory risk and an increased incidence of major cardiovascular events, indicating that the anti-inflammatory efficacy of statins is limited in practice (51, 52). *In vivo*, gemilukast showed greater anti-inflammatory activity than lovastatin, together with a more favorable hepatic safety profile under the conditions tested. Considering its combined lipid-lowering and anti-inflammatory effects, gemilukast holds promise for superior management of cardiovascular disease in the future. Notably, this study focused on systemic lipid/inflammatory readouts and mechanistic validation in hyperlipidemia models and did not include direct evaluation of cardiac structure or function; therefore, the impact of gemilukast on cardiac endpoints remains to be determined. In addition, macrophage polarization and signaling were primarily characterized in RAW 264.7 cells, an immortalized murine macrophage line that may not fully recapitulate primary human macrophage responses. Accordingly, future studies should validate these findings in human macrophage systems, with confirmation of key readouts including cytokine profiles, polarization markers, and PI3K/AKT modulation.

## 5 Conclusion

In summary, gemilukast emerges as a dual-action candidate with lipid-lowering and anti-inflammatory activities. Our results demonstrate that gemilukast reduced serum lipid levels and attenuated inflammation *in vitro* and *in vivo* models of hyperlipidemia. Mechanistically, gemilukast lowers lipids by reducing the solubility of cholesterol in mixed micelles and thereby limiting its uptake. In parallel, it modulates PI3K/AKT signaling to drive macrophage polarization from M1 to M2, resulting in markedly reduced inflammatory cytokine release. Importantly, this work provides proof-of-concept evidence that a clinically developed leukotriene receptor antagonist can be repurposed as a bifunctional scaffold to concurrently target intestinal cholesterol absorption and macrophage-driven inflammation, highlighting a dual-pathway strategy to address cardiometabolic risk. Overall, the convergence of lipid-lowering and anti-inflammatory effects supports gemilukast as a promising lead for cardiometabolic risk modification in dyslipidemia-associated cardiovascular disease. These findings provide a rationale for exploring gemilukast as an adjunct to standard lipid-lowering therapy in hyperlipidemia with heightened inflammatory burden. Future work should incorporate tracer-based cholesterol balance studies, intestinal segment-specific assays, and pathway perturbation in primary human macrophages, alongside longer-term efficacy and safety assessments in additional species to inform translational development.

## Data Availability

The original contributions presented in the study are included in the article/supplementary material. Further inquiries can be directed to the corresponding author.
