# Supplementary material for: Identification of gemilukast as a bifunctional molecule with lipid-lowering and anti-inflammatory activities
Source: Front Immunol. 2026 Mar 4;17:1738104. doi: 10.3389/fimmu.2026.1738104 (PMC12995635; doi:10.3389/fimmu.2026.1738104)
Supplement: Supplementary file 1 [file DataSheet1.docx]

Supplementary Materials for

**Identification of gemilukast as a bifunctional molecule with lipid-lowering and anti-inflammatory activities**

Yuanyuan Liu^1^, Yixiao Wang^2^, Rufei Wang^2^, Qingshan Yang^3^, Guojun Pan^2^, Renshuai Zhang^2*^

^1^Department of Internal Neurology, The First Affiliated Hospital of Jinzhou Medical University; Jinzhou, 121001, P. R. China.

^2^Shandong First Medical University & Shandong Academy of Medical Sciences; Jinan, 250117, P. R. China.

^3^Department of Radiation Oncology, The First Affiliated Hospital of Jinzhou Medical University; Jinzhou, 121001, P. R. China.

*Email: rszhang@sdfmu.edu.cn


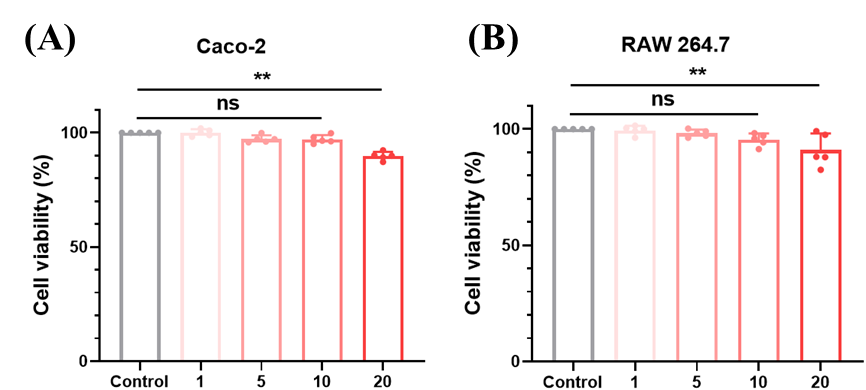


Figure S1. Gemilukast shows minimal cytotoxicity in Caco-2 and RAW 264.7 cells at the tested concentrations. The cell viabilities of Caco-2 cell (A) and RAW 264.7 macrophages (B) treated with gemilukast. Dots indicate independent replicates (n = 5). ^**^ *P* < 0.01; ns, not significant.


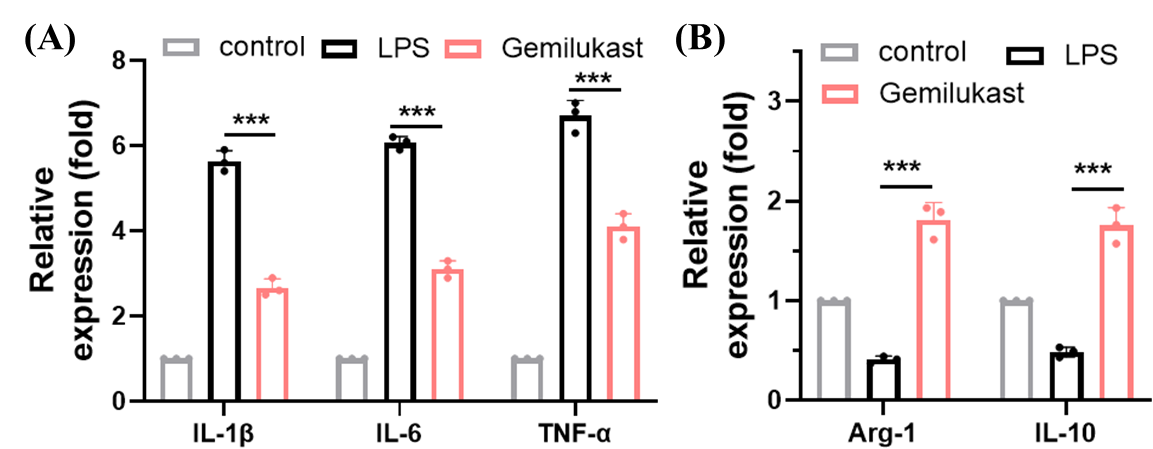


Figure S2. Gemilukast modulates macrophage polarization–related gene expression in RAW 264.7 cells. (A) The mRNA levels of M1 macrophage markers (IL-1β, IL-6 and TNF-α). (B) The mRNA levels of M2 macrophage markers (Arg-1 and IL-10). Dots indicate independent replicates (n = 3). ^***^*P* < 0.001.


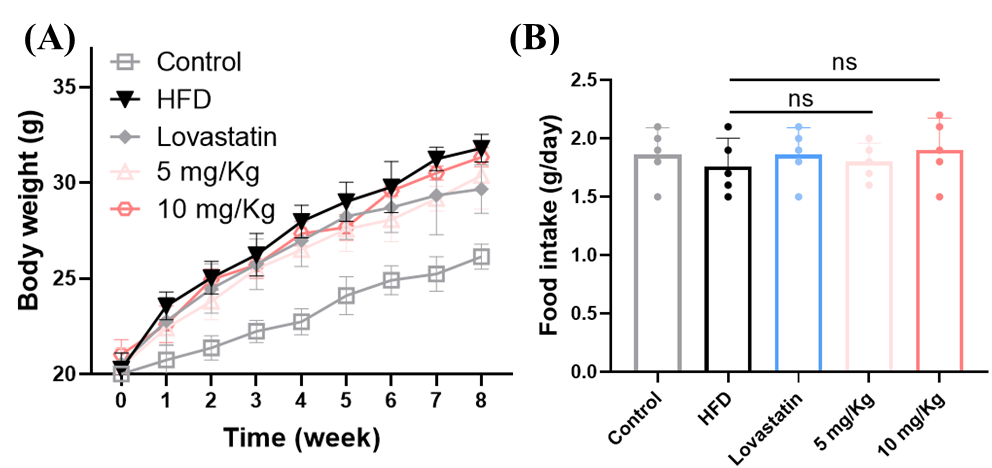


Figure S3. Body weight trajectory and food intake in the HFD study. (A) The curve of weight change over time within the experimental period. (B) Average daily food intake (g/day). ns, not significant.

Table S1. Primer sequences used for real-time PCR.

| Gene | Forward Primer (5′ to 3′) | Reverse Primer (5′ to 3′) |
| --- | --- | --- |
| IL-1β | ATGAAGGGCTGCTTCCAAAC | TCTCCACAGCCACAATGAGT |
| IL-6 | GGAGCCCACCAAGAACGATA | ACCAGCATCAGTCCCAAGAA |
| TNF-α | CTCATGCACCACCATCAAGG | ACCTGACCACTCTCCCTTTG |
| Arg-1 | TGGCTTGCGAGACGTAGAC | GCTCAGGTGAATCGGCCTTT |
| IL-10 | CTGGACAACATACTGCTAACCG | GGGCATCACTTCTACCAGGTAA |
| GAPDH | GGGTCCCAGCTTAGGTTCAT | CCAATACGGCCAAATCCGTT |
